# Effects of 6-Weeks High-Intensity Interval Training in Schoolchildren with Insulin Resistance: Influence of Biological Maturation on Metabolic, Body Composition, Cardiovascular and Performance Non-responses

**DOI:** 10.3389/fphys.2017.00444

**Published:** 2017-06-29

**Authors:** Cristian Alvarez, Rodrigo Ramírez-Campillo, Robinson Ramírez-Vélez, Mikel Izquierdo

**Affiliations:** ^1^Department of Physical Activity Sciences, Universidad de Los LagosOsorno, Chile; ^2^Research Nucleus in Health, Physical Activity and Sports, Universidad de Los LagosOsorno, Chile; ^3^Centro de Estudios en Medición de la Actividad Física, Escuela de Medicina y Ciencias de la Salud, Universidad del RosarioBogotá, Colombia; ^4^Department of Health Sciences, Public University of Navarre, CIBER de Fragilidad y Envejecimiento SaludableTudela, Spain

**Keywords:** interindividual variability, biological maturation, diabetes, performance, high-intensity interval training

## Abstract

**Background:** Previous studies have observed significant heterogeneity in the magnitude of change in measures of metabolic response to exercise training. There are a lack of studies examining the prevalence of non-responders (NRs) in children while considering other potential environmental factors involved such as biological maturation.

**Aim:** To compare the effects and prevalence of NRs to improve the insulin resistance level (by HOMA-IR), as well as to other anthropometric, cardiovascular, and performance co-variables, between early (EM) and normal maturation (NM) in insulin-resistance schoolchildren after 6-weeks of HIIT.

**Methods:** Sedentary children (age 11.4 ± 1.7 years) were randomized to either HIIT-EM group (*n* = 12) or HIIT-NM group (*n* = 17). Fasting glucose (FGL), fasting insulin (FINS) and homeostasis model assessment of insulin resistant (HOMA-IR) were assessed as the main outcomes, as well as the body composition [body mass, body mass index (BMI), waist circumference (WC), and tricipital (TSF), suprailiac (SSF) and abdominal skinfold (AbdSF)], cardiovascular systolic (SBP) and diastolic blood pressure (DBP), and muscular performance [one-repetition maximum strength leg-extension (1RM_LE_) and upper row (1RM_UR_) tests] co-variables were assessed before and after intervention. Responders or NRs to training were defined as a change in the typical error method from baseline to follow-up for the main outcomes and co-variables.

**Results:** There were no significant differences between groups in the prevalence of NRs based on FGL, FINS, and HOMA-IR. There were significant differences in NRs prevalence to decrease co-variables body mass (HIIT-EM 66.6% vs. HIIT-NM 35.2%) and SBP (HIIT-EM 41.6% vs. HIIT-NM 70.5%). A high risk [based on odds ratios (OR)] of NRs cases was detected for FGL, OR = 3.2 (0.2 to 5.6), and HOMA-IR, OR = 3.2 (0.2 to 6.0). Additionally, both HIIT-EM and HIIT-NM groups showed significant decreases (*P* < 0.05) in TSF, SSF, and AbdSF skinfold, and similar decreases in fasting insulin and HOMA-IR. The HIIT-EM group showed significant decreases in SBP. The HIIT-NM group showed significant increases in 1RM_LE_ and 1RM_UR_. A large effect size was observed for pre-post changes in TSF in both groups, as well as in SSF in the HIIT-NM group.

**Conclusion:** Although there were no differences in the prevalence of NRs to metabolic variables between groups of insulin resistance schoolchildren of different maturation starting, other NRs differences were found to body mass and systolic BP, suggesting that anthropometric and cardiovascular parameters can be playing a role in the NRs prevalence after HIIT. These results were displayed with several metabolic, body composition, blood pressure, and performance improvements independent of an early/normal maturation or the prevalence of NRs.

## Introduction

The benefits of exercise on health and performance are mainly expressed in terms of the “mean,” but there is wide interindividual variability in response to exercise training (IVRET) that has not been fully clarified in adults (Sisson et al., 2009; Bouchard et al., 2012; Álvarez et al., 2017b; Montero and Lundby, 2017) and not explored in children. IVRET means that under the same stimulus, some subjects may achieve positive benefits after training (i.e., responders – R), while others exhibit a worsened or unchanged response and are thus termed non-responders (NRs) (Bonafiglia et al., 2016). For example, although the mean of a group may indicate decreased fasting glucose after training, individuals in this group could show no changes or a worsened response and would thus be considered as NRs in terms of fasting glucose (Alvarez et al., 2017a) (in press Frontiers). Studies using endurance and high-intensity interval training (HIIT) in adults have described the occurrence of IVRET in performance variables such as maximum aerobic power (Prud'homme et al., 1984), maximum oxygen consumption (Vo_2_max) (Bouchard and Rankinen, 2001), and heart rate (Astorino and Schubert, 2014). More recently, other authors have described that under the same HIIT regime and under different health status conditions, there is different prevalence of NRs (i.e., percentage of NRs cases) to decrease both fasting glucose and fasting insulin metabolic variables in adults (Alvarez et al., 2017a).

Previous experimental trials have shown high-intensity protocols consisting of 8–10 1-min bouts of high-intensity exercise to be effective at improving both insulin and glucose parameters in adolescents (Bond et al., 2015; Cockcroft et al., 2015, 2017). However, despite of insulin resistance increases with age, racial health disparities and pubertal status (Ball et al., 2006), some authors have shown in girls and boys from 9 to 16 years an interesting relationship between physical activity and decreases in both insulin and the homeostasis model assessment of insulin resistance (HOMA-IR) marker changes throughout development (Metcalf et al., 2015).

On the other hand, age/pubertal status has been reported to be highly associated with more overweight/obesity (Wang, 2002). Analyses from the HELENA study showed high discrepancies between chronological and biological age in cardiorespiratory and strength performance (Ortega et al., 2008). Similarly, clear differences between those who were middle-prepubertal vs. late-pubertal have been observed regarding the level of metabolic substrate used during exercise (Stephens et al., 2006). It is unclear whether the metabolic benefits of exercise training are limited to those insulin-resistance children with early (EM) and normal (NM) initiation of biological maturation. To the best of the author's knowledge, the role of early maturation and the prevalence of NRs after a short-term HIIT intervention in children with insulin resistance is limited. Thus, despite that maturity has not showed a clear role on performance (Marta et al., 2014) in children, and considering that there is a lack of studies in glucose control parameters including the IVRET topic, It could, therefore, be suggested that one responsible of the chronic effects of exercise on glucose and insulin are dependent on age/pubertal status (Chu et al., 2014), with adolescents having a greater scope for improvements compared to younger children.

Thus, our objective was to compare the effects and prevalence of NRs to improve the insulin resistance level (by HOMA-IR), as well as to other anthropometric, cardiovascular, and performance co-variables, by early (EM) or normal maturation (NM) in insulin resistance schoolchildren after 6-weeks of HIIT. We hypothesized that regardless of biological maturation, there would be no difference in the prevalence of NRs according to level of glycemic control as defined by fasting glucose, fasting insulin and HOMA-IR in insulin-resistance children after a short HIIT regimen.

## Materials and methods

### Study design

This study was designed to address the question of how a school-based HIIT program affects NRs prevalence as defined by improved metabolic glucose control [fasting glucose, fasting insulin, and HOMA-IR], as well as other anthropometric, cardiovascular, and performance co-variables independent of different biological maturation stages in schoolchildren with insulin resistance. To accomplish this, we screened children at school to detect those insulin-resistance subjects, and after a short HIIT program, we compared the effects of 6-weeks of HIIT in 2 groups of children with different start times of biological sexual maturation: earlier (EM) and normal (NM) maturation starting.

### Participants

Initially, 150 schoolchildren (aged between 8 and 13 years), both boys and girls, with no background of regular HIIT volunteered to participate in this study. The eligibility criteria included the following: (a) age between 8 and 13 years [to include children with the capacity to follow the exercise instructions]; (b) address in an urban area; (c) diagnosis of insulin resistance in the screening applied at school (≤ 3 months) according to one of three glucose control markers: HOMA-IR ≥ 2.6 (following the cut-off point of a similar Chilean cohort of children) (Burrows et al., 2015), fasting insulin levels > 15 μU/dL (Reaven et al., 1993), or fasting glucose > 100 and <126 mg/dL (WHO, 1999); (d) physical inactivity (≤ 60 min/day of moderate physical activity) (O'Donovan et al., 2010); and (e) participation in the normal physical education class each week. The exclusion criteria included participants with: (a) potential medical problems or history of a familial metabolic disease such as T2DM in parents, (b) ischemic disease, (c) arrhythmia, (d) asthma, (e) chronic obstructive pulmonary disease, or (f) utilization of drugs that modulate metabolic and respiratory control.

In the 1st stage (enrollment stage), 106 subjects were not included for multiple reasons among subjects with both early and normal maturation: (a) age <8 years or > 13 years, (b) direct familial history of T2DM, (c) diagnosed asthma, (d) participation in regular physical activity, (e) address in a rural areas or (f) no diagnosed criterion of insulin resistance. Subsequently, 44 subjects, including those with EM and normal maturation (NM), were identified with insulin resistance at screening and were allocated into 2 groups: a HIIT early maturation group (HIIT-EM, *n* = 25) and a HIIT normal maturation group (HIIT-NM, *n* = 21). Thus, the final sample analyzed was as follows: HIIT-EM, age 11.0 ± 1.0 y, BMI 26.2 ± 5.6, *n* = 12 and HIIT-NM, age 12.0 ± 1.0 y, BMI 27.0 ± 4.7, *n* = 17. Subjects with <70% training attendance were excluded from all statistical analyses.

**Figure 1:**
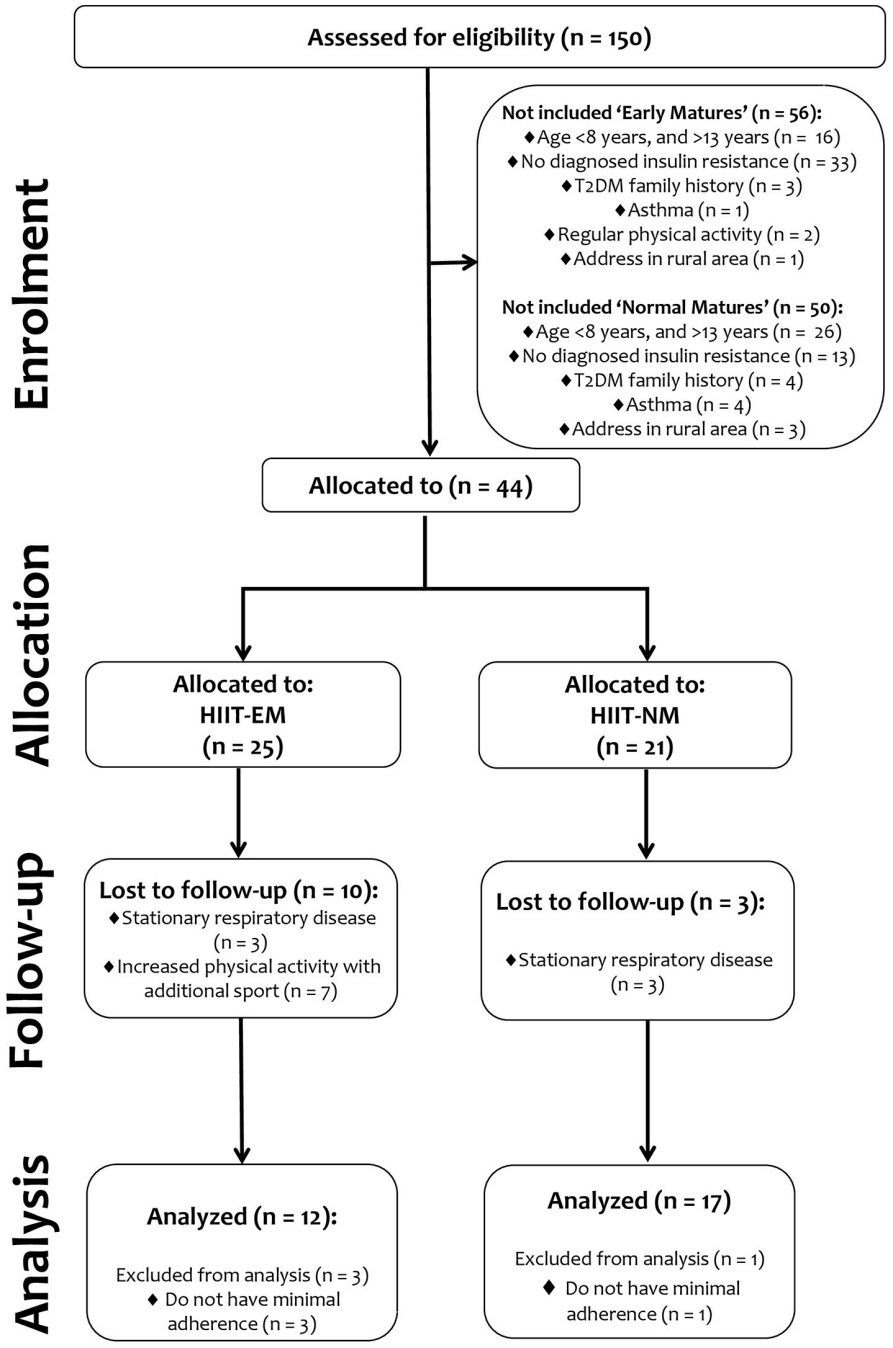
Study design.

Participants (and their parents/guardians) were informed of the experimental procedures at a meeting with the research team and were informed about the possible risks and benefits associated with participation in the study. Informed consent was also obtained at this meeting and before any of the assessments were performed. The study was conducted in accordance with the Declaration of Helsinki and was approved by the institutional review board for studies with human subjects of the local Ethics Committee of the University of Los Lagos (Comité de Revisión Científica y Ética Institucional del Departamento de Ciencias de la Actividad Física de la Universidad de Los Lagos). The sample size was computed according to the delta changes observed in the fasting glucose (ρFGL = 2.3; SD = 1.7 mg/dL) of a group that underwent a similar intervention (Álvarez et al., 2012). A statistical power analysis revealed that a total of 12 participants per group would yield a power of 80% at a 0.05 alpha level. The procedures were established according to the “CONSORT” statement, which can be found at http://www.consort-statement.org. Further details regarding the sample are presented in Figure 1.

During the 1st and 2nd week, participants were familiarized with the test procedures in 6 sessions (2 theoretical classes about the exercise procedures and 4 sessions to practice HIIT), before the initial assessment to understand the machines and weights as well as the protocols of the test. In the 3rd week before the performance measurements, subjects did not engage in any additional exercise training other than their regular physical education class (2 classes of 90 min), as this would disrupt the HIIT scheme. In week 4, the measurements were conducted in the following order: plasma samples were drawn in the morning and anthropometric assessments were performed in the afternoon (Monday). After 48 h, blood pressure measurements were taken, and tests of strength performance occurred in the morning and endurance performance in the afternoon (Wednesday and Friday). The measurements were completed in 5 days following the same order at the same time by the same professionals. In the 5th week, to reduce the potential effect of cumulative fatigue on dependent variables before and after the intervention, subjects had 7 days of rest between the last training and the first measurement session. Participants were instructed to wear similar athletic clothes during all testing sessions, as well as to drink water (not tea, coffee, or sugar meals) before and after training for hydration. Before the screening and allocation at school, 5 weeks were necessary to complete all the assessments; the last 2 weeks being used in the familiarization process, and the intervention was thus started in week 6. Before the intervention, there were significant baseline differences between groups in the dependent variables of genital maturation, height, and body mass. Other than these variables, there were no significant differences between groups in baseline characteristics (Tables 1, 2).

**Table 1:** Biological maturation characteristics and anthropometric pre-post changes of the subjects.

**Variable**	**Test**	**HIIT-EM (*n* = 12)**	**Effect size**	**HIIT-NM (*n* = 17)**	**Effect size**	***P*-value HIIT-EM vs. HIIT-NM baseline**	***P*-value HIIT-EM vs. HIIT-NM pre-post**
Gender (♀ / ♂)		7 / 4		9 / 8			
Age (y)	Pre	11.0 ± 1.0		12.0 ± 1.0		0.067	
Genital maturation	Pre	1.9 ± 1.0		2.9 ± 0.9		**<0.01**	
Pubic hair maturation	Pre	3.0 ± 1.0		3.0 ± 1.0		0.229	
Height (cm)	Pre	145.0 ± 0.11		153.0 ± 0.06		**<0.01**	**<0.01**
	Post	146.3 ± 0.06		154.6 ± 0.09			
**ANTHROPOMETRIC**							
Body mass index (kg/m^2^)	Pre	26.2 ± 5.6	0.02 (−0.07, 0.10)	27.1 ± 4.7	−0.08 (−0.14, −0.02)	0.633	**0.023**
	Post	26.3 ± 5.4		26.7 ± 4.2			
	*P*-value	0.322		0.065			
	Δ%	0.3		−1.4			
Waist circumference (cm)	Pre	86.8 ± 14.0	−0.01 (−0.31, 0.04)	88.0 ± 9.8	−0.22 (−0.35, −0.10)^$^	0.801	0.345
	Post	84.6 ± 13.2		85.7 ± 9.1			
	*P*-value	0.144		0.139			
	Δ%	−2.5		−2.6			
Supra-iliac skinfold (mm)	Pre	42.5 ± 10.8	−0.44 (−0.60, −0.29)^$^	41.1 ± 6.7	−1.24 (−1.55, −0.92)^‡^	0.661	0.056
	Post	35.7 ± 9.0		33.3 ± 5.8			
	*P*-value	**<0.01**		**<0.01**			
	Δ%	−16.0		−18.9			

*Data presented as mean and ± SD. Delta changes (Δ%) is presented in percentage. Groups are described as HIIT-EM, high intensity interval training earlier matures; HIIT-NM, high intensity interval training normal matures. Variables are described as ♀ = girls; ♂ = boys. Bold values indicate significant differences at level P ≤ 0.05*.

$ *Small standardized effect at level P ≤ 0.05*.

‡ *Large standardized effect at level P ≤ 0.05*.

**Table 2:** Characteristics and pre-post changes of the subjects at level of blood pressure, metabolic, and performance variables.

**Variable**	**Test**	**HIIT-EM (*n* = 12)**	**Effect size**	**HIIT-NM (*n* = 17)**	**Effect size**	***P*-value HIIT-EM vs. HIIT-NM baseline**	***P*-value HIIT-EM vs. HIIT-NM pre-post**
**PERFORMANCE**							
1RM_LE_ (kg)	Pre	14 ± 6	0.67 (0.22, 1.12)^§^	19 ± 6	0.92 (0.36, 1.48)^§^	0.089	0.678
	Post	20 ± 8		27 ± 6			
	*P*-value	0.060		**<0.001**			
	Δ%	+42.8		+42.1			
1RM_UR_ (kg)	Pre	6 ± 2	0.89 (0.27, 1.51)^§^	8 ± 3	0.58 (0.11, 1.06)^$^	0.114	0.334
	Post	8 ± 3		10 ± 4			
	*P*-value	0.093		**<0.001**			
	Δ%	+33.3		+25.0			

*Data presented as mean and ±SD. Delta changes (Δ%) is presented in percentage. Groups are described as HIIT-EM, high intensity interval training earlier matures; HIIT-NM, high intensity interval training normal matures. Variables are described as 1RM_LE_ = 1 maximum repetition leg-extension; 1RM_UR_ = 1 maximum repetition upper row. Bold values denotes significant pre-post changes intra group*.

$ *Small standardized effect at level P ≤ 0.05*.

§ *Moderate standardized effect at level P ≤ 0.05*.

### Classification of responders (R) and non-responders (NRs)

Following previous criteria applied in exercise interventions (Bonafiglia et al., 2016), the IVRET of the subjects was categorized into responders (R) and non-responders (NRs) using the typical error method (TE). The TE was calculated for the main outcomes (fasting glucose, fasting insulin and HOMA-IR), as well as for the other anthropometric, cardiovascular, and performance co-variables, as described previously (Álvarez et al., 2017b), using the following equation:

$$\begin{array}{l} TE=SD_{diff}/\surd 2 \end{array}$$

where *SD*_*diff*_ is the variance (standard deviation) in the difference in scores observed between the 2 repeats of each test. The NRs to decrease fasting glucose, fasting insulin, and HOMA-IR, as well as in all the co-variables, were defined as those individuals who failed to demonstrate an increase or decrease (in favor of beneficial changes) greater than 2 times the TE away from zero. A change 2 times greater than the TE indicated a high probability (i.e., 12 to 1 odds) that the response was a true physiological adaptation beyond what might be expected from technical and/or biological variability (Hopkins, 2000).

### Classification of the start of biological maturation

Subjects' biological maturation was classified using a self-reported personal questionnaire that assessed Tanner stages (pubic hair stages for both sexes, breast stage for girls, and genitalia stage for boys) that has previously been used (Matsudo and Matsudo, 1994). The subjects were briefly informed about the questionnaire by a specialist. A male subject was classified in the “early maturation” group if the development of his genitalia was scored as stage 2 and his chronological age was less than the average age of the sample with genitalia stage 2; we previously calculated the average age of individuals in each Tanner stage (1 to 5) in boys and girls (Wang, 2002). This classification allowed us to identify the chronological age when a child was in “mean biological maturation” or was younger than this value (i.e., early maturation) according to each Tanner stage.

### Metabolic measurements

Subjects arrived with their parents to the Riñihue's clinic between 08.00 and 10.00 in the morning after 10 h of overnight fasting, and blood samples (3.5 mL) were collected in tubes with specific anticoagulant gel to collect glucose and insulin. Samples were immediately placed on ice and centrifuged at 4,000 rpm (1,700 × g) for 5 min at 4°C. Plasma samples were immediately transferred to pre-chilled microtubes and stored at −20°C for later analysis. Plasma glucose was analyzed by enzymatic methods using standard kits (Wiener Lab Inc., Rosario, Argentina) with an automatic analyzer (Metrolab 2300 Plus™, Metrolab Biomed Inc., Buenos Aires, Argentina). Fasting insulin was measured by RIA (DPC, Los Angeles, CA, USA). The HOMA-IR index was calculated using the Matthews equation (Matthews et al., 1985): insulin resistance = [glucose (mg/dL) × insulin (μU/dL)] / 405). The same blood sampling and preparation procedures were performed at the end of the 6-weeks follow-up 48 h after the last exercise session to avoid the possible acute effects of exercise.

### Anthropometric measurements

Anthropometric measurements were taken after plasma blood sampling, 3 days before the performance measurements. Body mass (in kilograms) was assessed using an electrical bio-impedance scale with 0.1 kg accuracy (Omron HBF-INT™, Omron Healthcare Inc., Lake Forest, IL, USA), similar to other studies (Corte de Araujo et al., 2012). Standing height (in centimeters) was assessed with a professional stadiometer (Health o Meter™ Professional, Sunbeam Products Inc., Chicago, IL, USA) to an accuracy of 0.1 cm, and BMI was calculated (kg/m^2^). Waist circumference was assessed with an inextensible measuring tape with 0.1 cm accuracy (Hoechstmass^TM^, Sulzbach, Germany). Additionally, 3 skinfold measurements of subcutaneous adipose tissue (tricipital, suprailiac, and abdominal skinfold) were assessed using a Langue™ skinfold caliper (Beta Technology Inc., Santa Cruz, California, USA) according to standard protocols (Marfell-Jones et al., 2006).

### Muscle performance measurements

Muscle performance tests were conducted as previously reported (Faigenbaum et al., 1996). The one-repetition maximum strength tests were performed to two exercise; leg extension (1RM_LE_) using an exercise machine (OXFORD^TM^, model EE4002, Santiago, Chile) and 1RM of an upper row (1RM_UR_) test using weights and metal bars. The highest load of three attempts per exercise was recorded. The test procedure was repeated at the same time and in the same order as the post-intervention measurement by the same evaluator, who was blinded to subject's group assignments.

### HIIT

A total of 18 sessions (3 times per week) of the HIIT program were conducted. Cycle ergometers adapted for children (OXFORD^TM^, model BE2601, OXOFORD Inc., Santiago, Chile) were used. Each participant performed a range of 8 to 12 cycling intervals (weeks 1–2; 8, weeks 3–4; 10, weeks 5–6; 12 intervals) during the intervention period. The duration of each cycling interval increased progressively each week and ranged between 40 and 60 s (40 s weeks 1–2; 50 s weeks 3–5; 60 s week 6), with 120 s of passive rest (on the bicycle without movement) between each work interval. Cycle revolutions were determined at a range of 50–70 revolutions per min (rpm) and a speed between 20 and 40 km/h during each work interval.

The modified Borg scale (RPE) was applied to assess subjective effort as a marker of intensity to guide the training, specifically to maintain a score between 8 and 10 RPE points during each cycling interval (Ciolac et al., 2015), and the cycle ergometer load was adjusted every ~2 weeks to maintain this subjective intensity during cycling. This subjective intensity corresponded to a range of 70 to 100% of maximum heart rate according to the Karvonen formula (Karvonen and Vuorimaa, 1988). A professional physiologist provided the instructions to start each work interval during the sessions. Each training session was performed in the afternoon from 4 to 6 pm throughout the 6-week period and was closely monitored by exercise physiologists (Behm et al., 2008). All subjects had good exercise tolerance, and none of the participants reported an injury. The exercise compliance was 80.0 ± 1% in the HIIT-EM group and 94.4 ± 3% in the HIIT-NM group during the follow-up. Characteristics of the training sessions are presented in (Table 3).

**Table 3:** Characteristics of the HIIT training.

**Variable**	**Week 1**	**Week 2**	**Week 3**	**Week 4**	**Week 5**	**Week 6**
Duration of interval of work (s)	40	40	50	50	60	60
Duration of interval of rest (s)	120	120	120	120	120	120
Number of intervals of work (N°)	8	8	10	10	12	12
Number of intervals of rest (N°)	9	9	11	11	13	13
Qualitative intensity in Borg scale 1–10 (pts)	8–10	8–10	8–10	8–10	8–10	8–10
Quantitative intensity by heart rate (%)	70–100	70–100	70–100	70–100	70–100	70–100
Cadence (rpm)	50–70	50–70	50–70	50–70	50–70	50–70
Velocity (km/h)	20–40	20–40	20–40	20–40	20–40	20–40
Volume of work / session (min)	5.3	5.3	8.3	8.3	14	14
Volume of work / week (min)	15.9	15.9	24.9	24.9	42	42
Volume of rest / session (min	18	18	22	22	26	26
Volume of rest / week (min)	54	54	66	66	78	78
Total time investment / session (min)	23.3	23.3	30.3	30.3	40.0	40.0
Total time investment / week (min)	69.9	69.9	90.9	90.9	120.0	120.0
Total time investment / 6-weeks (h)	–	–	–	–	–	4.68

*s, seconds; N°, numbers; pts, points; %, percentage; rpm: revolutions per minute; km/h, kilometers per hour; min, minutes; h, hours*.

### Statistical analysis

Data are presented as mean ± standard deviation (SD). Assumptions of normality and homoscedasticity for all data were checked using the Shapiro-Wilk and Levene tests, respectively. The Wilcoxon test was used for non-parametric data (waist circumference, systolic BP, and 1RM_UR_). One-way ANOVA was conducted to test for baseline differences between groups. ANCOVA was performed to assess differences in baseline body mass using WC and the 3 skinfold measurements as co-variables. A repeated-measures ANOVA with 2 factors (group × time) was used to determine the differences in all dependent variables between the pre- and post 6-weeks tests using each group × time interaction. After the intervention, delta values (Δ) in percentages (%) were calculated between pre- and post-intervention assessments of fasting glucose, fasting insulin, and HOMA-IR. Subjects were categorized as responders (R) or non-responders (NRs) using the typical error (TE) method for each dependent variable according to the previously described criteria of 2 TE (Bonafiglia et al., 2016). Bonferroni *post hoc* test was applied to establish the differences between groups. Additionally, Cohen's test was used to detect the effect size (*d*), with threshold values of 0.20, 0.60, 1.2, and 2.0 for small, moderate, large, and very large effects, respectively (Hopkins et al., 2009). To test for differences between NRs by HIIT-EM x HIIT-NM groups, chi-square test (*X*^2^) was used for categorical variables. The odds ratios (OR) of being a non-responder were calculated for the differences in dichotomous NRs variables between groups. All statistical analyses were performed with SPSS statistical software version 18 (SPSS® Inc., Chicago, Illinois, USA). The alpha level was fixed at *P* ≤ 0.05 to indicate statistical significance.

## Results

### Baseline differences

Before training, there were significant differences (*P* < 0.05) between groups in genital maturation, height, and body mass (Table 1).

### Training-induced changes

**Figure 2:**
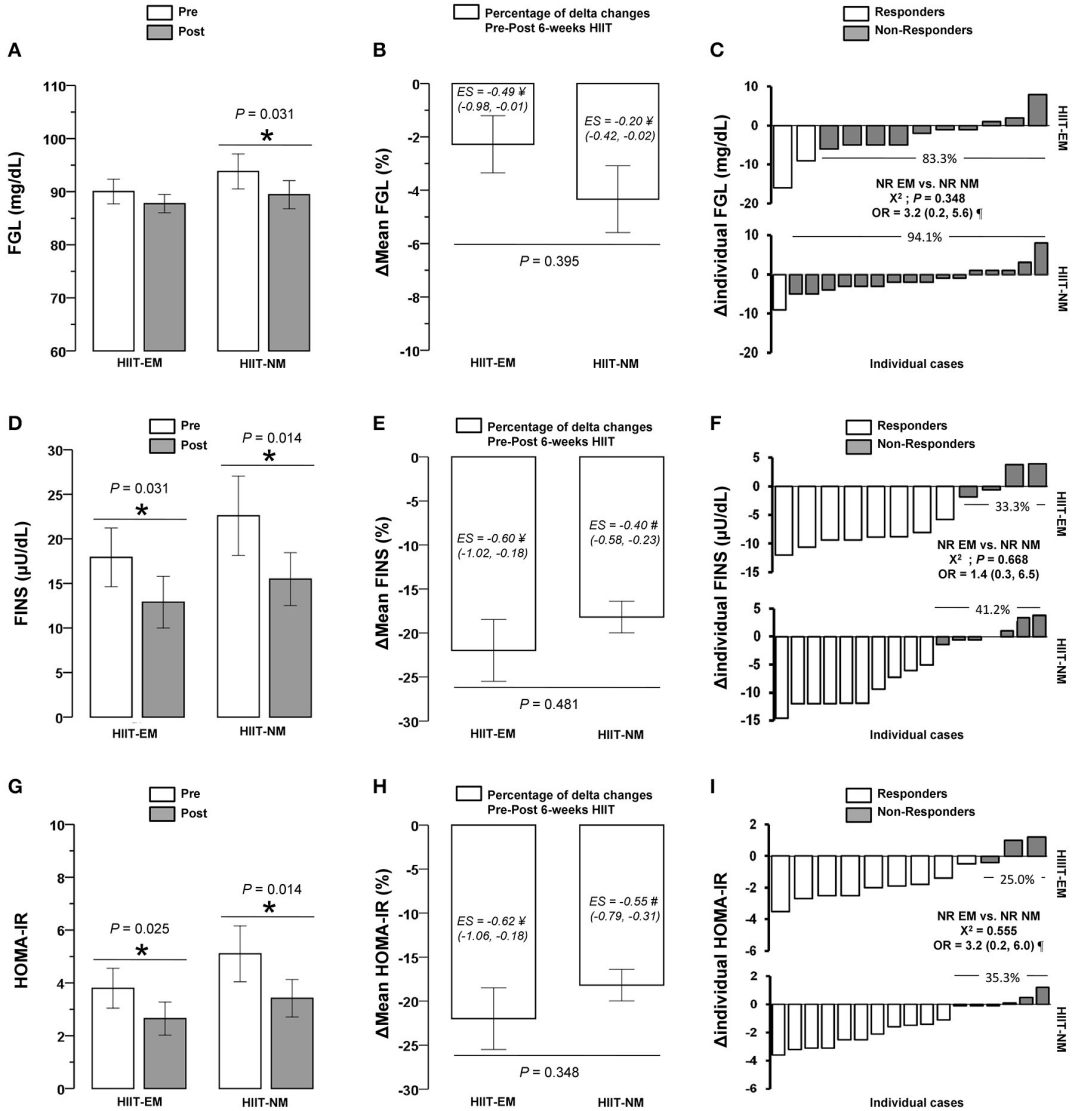
Pre-post changes **(A,D,G)**, delta percent (mean) **(B,E,H)**, and delta (individual) **(C,F,I)** of body mass, tricipital, and abdominal skinfold after 6 weeks of HIIT in insulin-resistance schoolchildren. Groups are described as: HIIT-EM, high-intensity interval training early maturation group; HIIT-NM, high-intensity interval training normal maturation group. ^*^Denotes significant pre-post intragroup changes at level *P* < 0.05. ^†^Denotes significant differences between HIIT-EM vs. HIIT-NM groups at level *P* < 0.05. ^#^Denotes ‘small’ statistical effect size at *P* < 0.05. ^¥^Denotes ‘moderate’ statistical effect size at *P* < 0.05. ^&^Denotes ‘large’ statistical effect size at *P* < 0.05. ^¶^Denotes high risk of suffering a non-response.

**Figure 3:**
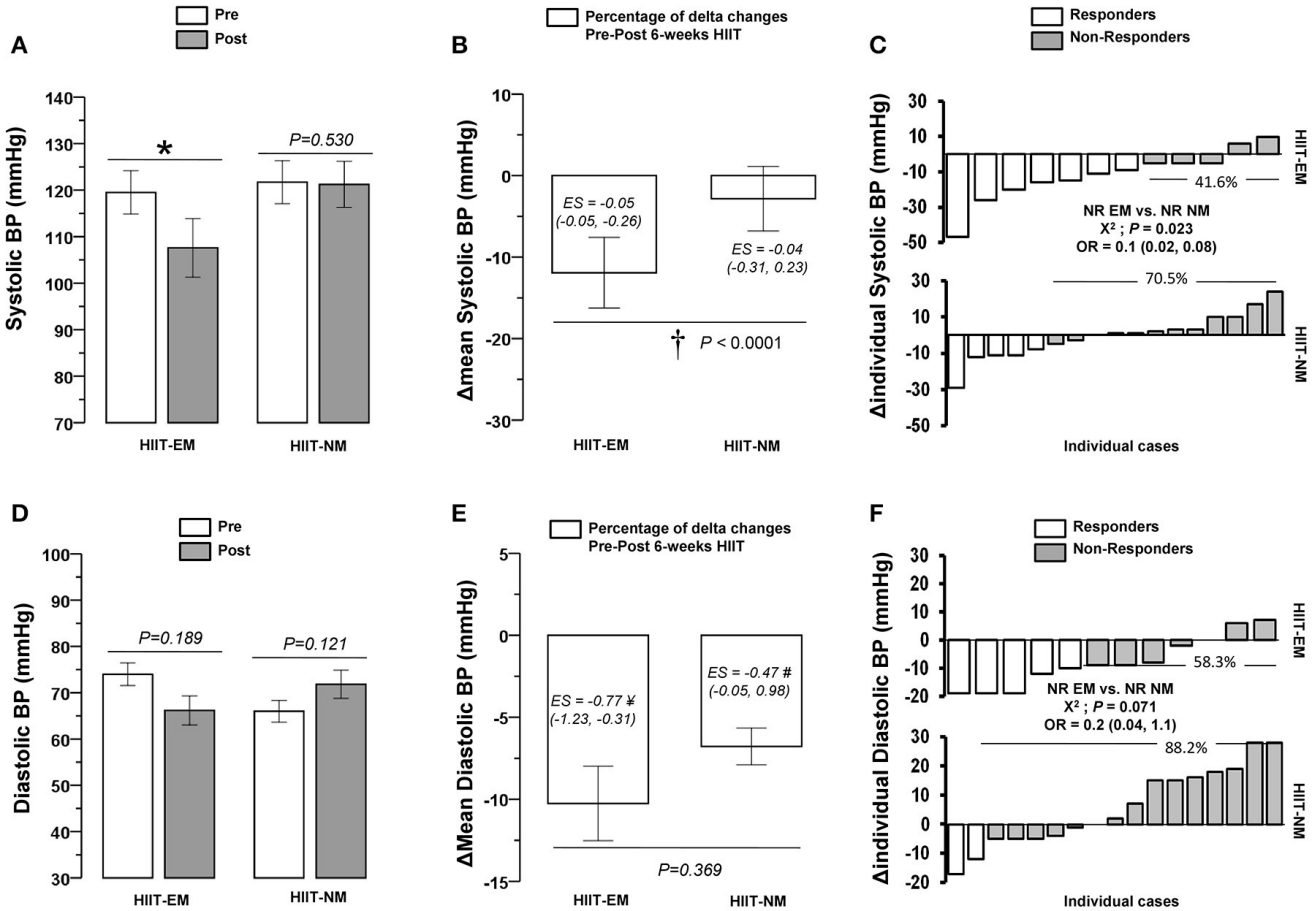
Pre-post changes **(A,D)**, delta percent (mean) **(B,E)**, and delta (individual) **(C,F)** of systolic and diastolic blood pressure after 6 weeks of HIIT in insulin-resistance schoolchildren. ^*^Denotes significant pre-post intragroup changes at level *P* < 0.05. ^†^Denotes significant differences between HIIT-EM vs. HIIT-NM groups at level *P* < 0.05. ^#^Denotes ‘small’ statistical effect size at *P* < 0.05. ^¥^Denotes ‘moderate’ statistical effect size at *P* < 0.05. ^&^Denotes ‘large’ statistical effect size at *P* < 0.05. ^¶^Denotes high risk of suffering a non-response.

**Figure 4:**
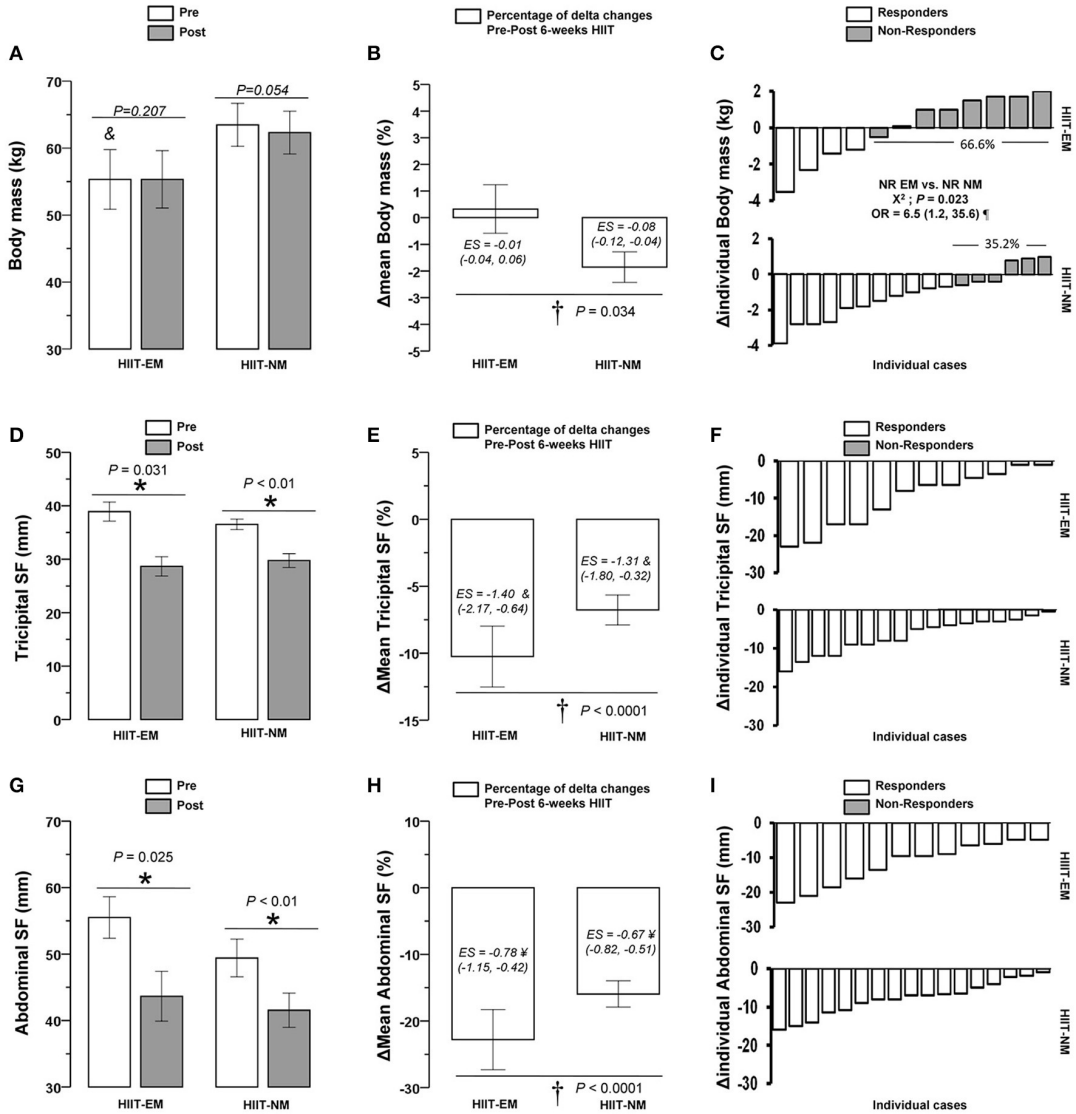
Pre-post changes **(A,D,G)**, delta percent (mean) **(B,E,H)**, and delta (individual) **(C,F,I)** of fasting glucose, fasting insulin and homeostasis model assessment of insulin-resistance in insulin-resistance schoolchildren. FGL, fasting glucose, FINS, fasting insulin, HOMA-IR, homeostasis model assessment of insulin resistance. ^*^Denotes significant pre-post intragroup changes at level *P* < 0.05. ^†^Denotes significant differences between HIIT-EM vs. HIIT-NM groups at level *P* < 0.05. ^#^Denotes ‘small’ statistical effect size at *P* < 0.05. ^¥^Denotes ‘moderate’ statistical effect size at *P* < 0.05. ^&^Denotes ‘large’ statistical effect size at *P* < 0.05. ^¶^Denotes high risk of suffering a non-response.

After training, in the HIIT-EM group, no significant changes were observed in body mass (Figure 2), BMI and WC (Table 1), DBP (Figure 3D), FGL and 1RM_LE_ (Figure 4A, Table 2). In the HIIT-NM group, no significant changes were observed in body mass (Figure 2A), BMI and WC (Table 2), systolic/diastolic BP (Figures 2A,D) or FGL (Figure 4A). After training, in the HIIT-EM group, there were significant decreases in delta percent mean (ΔMean) in anthropometric variables, namely, TSF −10.3% and AbdSF −22.8% (Figures 2E,H, respectively), SSF −16.0% (Table 1), and systolic BP −11.9% (Figure 3B), and in metabolic variables, namely, FINS −22.8% and HOMA-IR −22.9% (Figures 4E,H, respectively). In the HIIT-NM group, there were significant changes in anthropometric variables, i.e., TSF −6.8% (Figure 2E), SSF −18.9% (Table 1), and AbdSF −15.9% (Figure 2H); metabolic variables, i.e., FINS −22.7% and HOMA-IR −15.8% (Figures 4E,H, respectively) and muscle performance variables, i.e., 1RM_LE_ +42.1% and 1RM_UR_ +25.0% (Table 2). A large statistical effect size was found for TSF in both the HIIT-EM (−1.40; 90% CI = −2.17, −0.64) and HIIT-NM group (−1.31; 90% CI = −1.80, −0.82) (Figure 2E), as well as for SSF in the HIIT-NM group (−1.24; 90% CI = −1.55, −0.92) (Table 1).

### Prevalence of non-responders in other anthropometric, cardiovascular, and muscle performance co-variables

There were significant differences (*P* < 0.05) in NRs prevalence between groups in terms of decreased body mass, (HIIT-EM group 66.6% vs. HIIT-NM group 35.2%), as well as decreased systolic BP (HIIT-EM group 41.6% vs. HIIT-NM group 70.5%) (Figure 3C).

There were no significant differences in the prevalence of NRs between groups in the other dependent co-variables tested (Table 4). There were no NRs based on TSF or AbdSF in either group (Figures 2F,I, respectively), including 1RM_LE_ in the HIIT-NM group (Table 4).

**Table 4:** Differences in the non-responders prevalence to improve anthropometric, and performance parameters in children with insulin resistance after 6-weeks of HIIT intervention.

**Variable**	**Response Type**	**HIIT-EM (*n* = 12)**	**HIIT-NM (*n* = 17)**	**OR (95% CI) for NRs**	***P*-value HIIT-EM vs. HIIT-NM**
Gender (♀ / ♂)		9 / 3	11 / 6		
**ANTHROPOMETRIC**					
Body mass index (% / *n* =)	NRs	66.7 (8)	35.3 (6)	3.6 (0.7 to 17.4) ¶	0.096
	R	33.3 (4)	64.7 (11)		
Waist circumference (% / *n* =)	NRs	33.3 (4)	35.3 (6)	0.9 (0.1 to 4.3)	0.913
	R	66.7 (8)	64.7 (11)		
Supra-iliac skinfold (% / *n* =)	NRs	8.3 (1)	5.9 (1)	1.4 (0.1 to 25.8)	0.798
	R	91.7 (11)	94.1 (16)		
**PERFORMANCE**					
1RM_LE_(% / *n* =)	NRs	8.3 (1)	0 (0)	2.5(1.6 to 4.0) ¶	0.226
	R	91.7 (11)	100 (17)		
1RM_UR_(% / *n* =)	NRs	41.7 (5)	35.3 (6)	1.3 (0.2 to 5.9)	0.728
	R	58.3 (7)	64.7 (11)		

*HIIT-EM, high-intensity interval training earlier matures group; HIIT-NM, high-intensity interval training normal matures group; 1RM_LE_, one maximum repetition strength test; 1RM_UR_, one maximum repetition upper row strength test; OR, odds Ratios; NRs, non-responders; R, responders*.

¶ *Denote high risk (≥2 fold) to suffer a NR in HIIT-EM vs. HIIT-NM group*.

The risk of being a NRs according to the OR was high (≥ 2-fold) for the variables body mass (OR = 6.5, 95% CI = 1.2, 36.6, *P* = 0.023) (Figure 2C), BMI (OR = 3.6, 95% CI = 0.7, 17.4, *P* = 0.096) and 1RM_LE_ (OR = 2.5, 95% CI = 1.6, 4.0, *P* = 0.728) (Table 4) in the HIIT-EM group vs. the HIIT-NM group.

### Prevalence of non-responders in terms of metabolic variables

There were no significant differences (*P* < 0.05) between the HIIT-EM and HIIT-NM groups in NRs prevalence according to decreased FGL (83.3% vs. 94.1%, respectively, *P* = 0.348) (Figure 4C), decreased FINS (33.3 vs. 41.2%, respectively, *P* = 0.668) (Figure 4F), or HOMA-IR (25 vs. 35.3%, respectively *P* = 0.555) (Figure 4I).

## Discussion

This study has four main results: (i) there were no significant differences between children with EM and NM in the prevalence of NRs based on improved metabolic profiles (fasting glucose, fasting insulin, HOMA-IR); (ii) independent of NRs prevalence and biological maturation (i.e., EM, NM), HIIT was able to decrease fasting insulin and HOMA-IR in insulin-resistance schoolchildren; (iii) there were significant differences in NRs prevalence in terms of anthropometric (body mass) and systolic BP co-variables; and (iv) HIIT promoted improvements in the body composition (decreased skinfold), decreased systolic BP, and muscular performance co-variables included in this study.

To the author's knowledge, there is no evidence regarding the prevalence of NRs after HIIT interventions in children with insulin resistance. In this study, HIIT was able to reduce subcutaneous fat in both the HIIT-EM and HIIT-NM groups including TSF −10.3 vs. −6.8%, SSF −16.0 vs. −18.9%, and AbdSF −22.8 vs. −15.9%, respectively (Figure 2, Table 1). These results are in accordance with previous HIIT interventions (1 min work interval, 3 min recovery, 3−6 bouts, 12 weeks) in children that reported a decrease in body mass of −2.7%, fat mass of −2.6%, and WC of −7% (Corte de Araujo et al., 2012). Unfortunately, these authors did not report the prevalence of NRs. Despite the fact that there are known differences in biological maturation between children of the same chronological age (Ortega et al., 2008), it remains unknown whether children with early or normal maturation are more commonly NRs to similar modes of training, such as HIIT. Among the unknown effects of HIIT on NRs prevalence, we found in this study that there were no NRs in terms of decreased TSF or AbdSF in both the early and normal maturation groups (Figure 2). In line with this, after 12-weeks of endurance training in adults, there was an NRs prevalence according to decreased body mass and body fat of 3.3 and 13.3%, respectively (King et al., 2008). After 9 months of HIIT (15−30 s, 2 bouts/10 min, at 80% of maximal aerobic power, treadmill/cycling), previous authors showed a 7.2% prevalence of NRs based on decreased WC and an 8.6% prevalence of NRs in decreased total fat mass among subjects with metabolic syndrome (Gremeaux et al., 2012). It is worth noting that only 2-weeks of HIIT in adults led to decreases in WC of −2.3% (Whyte et al., 2010). In addition, it appears that HIIT rapidly leads to benefits regarding improved anthropometric markers, such as skinfold measurements in children, and these findings are in accordance with studies in adults. Thus, it appears that in a sample size regularly used in exercise interventions (i.e., ~10 subjects) and with high compliance, HIIT has an important contribution to decreasing fat; this finding has been reported after HIIT regimens, in which adrenergic mechanisms post-exercise have an important role (Boutcher, 2011).

Additionally, we observed that the HIIT-NM group showed a higher prevalence of NRs in terms of decreased systolic BP, at 70.5%, than the HIIT-EM group, which showed a 41.6% prevalence of NRs (Figure 3C). Other studies have shown a NRs prevalence of 60.9% in decreased systolic BP and of 59.1% in decreased DBP after 5 months of endurance (65–80% Vo_2_peak, walking/jogging), strength (8–12 repetitions per set, 8 exercises, 70–85% of 1RM, 3 days/week), or concurrent training (Moker et al., 2014). Regarding the lack of evidence on the potential influence of the start of biological maturation on promoting more or less NRs in terms of glucose control among children in a HIIT regime at school, the earlier maturation group in this study apparently had a lower risk of NRs in terms of decreased systolic BP after this mode of training (Figure 3C). We found a significant decrease in systolic BP of −11.9% in the HIIT-EM group, which was greater than the decrease in the HIIT-NM group of −2.8%. We speculate that HIIT rapidly promotes angiogenic factors to increase capillarization, which provides an advantage that is translated to the limited muscle mass of the HIIT-EM group compared to that of children with normal maturation who apparently present more insulin resistance. Similarly, a NRs prevalence in decreased systolic BP of 12.2% was reported after endurance training (30–50 min/session, 3 days/week, 55–75% Vo_2_max, 20 weeks) in a study assessing a wide sample of subjects (Bouchard et al., 2012). Other HIIT-based studies in adults have reported a NRs prevalence in terms of decreased diastolic BP of 61.5%, and our study is in accordance with this finding, reporting a value of 58.8% (Higgins et al., 2015).

Subjects with both early and normal maturation showed decreases in FINS of −22.8% and −22.7% and in HOMA-IR of −22.9% and −15.8%, respectively, after the intervention (Figure 4), and there were no differences between groups in the prevalence of NRs in terms of decreased FGL, FINS or HOMA-IR (Figure 2). There were no cases of NRs in TSF or AbdSF (Figure 2) in either group or in 1RM_LE_ in the HIIT-NM group (Table 4), in which all subjects were responders. Thus, both TSF and AbdSF showed a high sensitivity to change after HIIT interventions. Some authors have reported a NRs prevalence of 8.4% in decreased FINS; however, these studies examined adults and the effects of endurance training (Bouchard et al., 2012). To the author's knowledge, there are no studies reporting the prevalence of NRs after HIIT in children or how earlier or normal maturation influences the response after training. Based on our results of decreases in HOMA-IR in both the early and normal maturation groups of −35.7 and −26.9%, respectively, we confirm that HIIT is a powerful mode of training for sedentary, insulin-resistance children and additionally that HIIT results in few or no NRs in studies including a standard sample size, such as this one. This suggests that neither an early nor a normal start of biological maturation plays a role in the NRs prevalence as measured by decreased FGL, FINS, or HOMA-IR in children with insulin resistance.

Moreover, we did not find differences in NRs prevalence based on increased 1RM_LE_ or 1RM_UR_ in either intervention group (Table 4). We also observed a high risk (≥2-fold) of being a NRs in the HIIT-EM, at 8.3%, vs. the HIIT-NM group at 0%, but this included only 1 case. We observed significant increases in 1RM_LE_ and 1RM_UR_ in the HIIT-NM group of +42.1 and +25.0%, respectively (Table 2). We can state in general that HIIT is able to increase the strength performance of lower limbs by cycling when the effort is tailored to 8–10 points on the modified Borg scale and the load progressively adjusted to maintain this qualitative short-term effort.

A strength of this study was that we included a sample of 10–20 subjects in each group, and this size is frequently used in training interventions (Ziemann et al., 2011). We also reported pre-post changes, as well as the effect size and OR of NRs for each group. Additionally, we assessed other anthropometric, cardiovascular, metabolic and muscle performance co-variables regularly used in training studies with children. One limitation was that we did not control for additional exercise after each training session, but this information was recorded each week in children and parents to maintain similar baseline conditions of exercise and diet. Additionally, among children, it is widely known that they have an increased energy expenditure, and we presume that part of the differences in the training-induced changes between subjects in the HIIT-EM vs. HIIT-NM groups were due to discrepancies in hormonal and molecular processes.

## Conclusion

In conclusion, although there were no differences in the prevalence of NRs to metabolic variables between groups of insulin resistance schoolchildren of different maturation starting, other NRs differences were found to body mass and systolic BP, suggesting that anthropometric and cardiovascular parameters can be playing a role in the NRs prevalence after HIIT. These results were displayed with several metabolic, body composition, blood pressure, and performance improvements independent of an early or normal maturation or the prevalence of NRs.

## Disclosure

The work described has not been published previously, it is not under consideration for publication elsewhere. The publication is approved by all authors. If accepted, it will not be published elsewhere in the same form, in English or in any other language, including electronically without the written consent of the copyright-holder. All authors have approved the final article should be true and included in the disclosure.

## Author contributions

CA conceived and designed the research project. CA and RRC reviewed the literature studies and conducted data extraction. CA conducted data analyses and fieldwork. CA, RRC, and MI were responsible for data interpretation. CA and RRC drafted the manuscript, and RRV and MI critically revised the manuscript for intellectual contributions. CA and RRC coordinated the study development. All authors reviewed and edited the manuscript. All authors read and approved the final manuscript.

### Conflict of interest statement

The authors declare that the research was conducted in the absence of any commercial or financial relationships that could be construed as a potential conflict of interest.
